# Multi-functional stretchable sensors based on a 3D-rGO wrinkled microarchitecture†

**DOI:** 10.1039/c9na00429g

**Published:** 2019-09-27

**Authors:** Jin Jia, Guotao Huang, Mingti Wang, Yuhuan Lv, Xiangyang Chen, Jianping Deng, Kai Pan

**Affiliations:** College of Materials Science and Engineering, Beijing University of Chemical Technology Beijing 100029 China

## Abstract

The structural design of sensing active layers plays a critical role in the development of electromechanical sensors. In this study, we established an innovative concept for constructing sensors, pre-straining & laser reduction (PS&LR), based on a laser-induced wrinkle effect. This method combines and highlights the advantages of a wrinkled structure in the flexibility of sensors and the advantages of laser in the efficient reduction of GO; thus, it can efficiently introduce tunable, stretchable 3D-rGO expansion bulges in wrinkled GO films. Particularly, the sensors based on this special structure (1.5 cm × 3 cm) demonstrated a multi-functional and distinguished sensing ability in the cases of bending, stretching and touching modes. Moreover, the 3D-rGO architecture endowed the sensors with great sensitivity and design flexibility, *i.e.*, a high sensing factor of 122, relative current value change of 60 times at the bending angle of 60°, decreased relative resistance–strain curve and diverse bending strategies for various detection purposes. Thus, the established design and preparation strategy provides large design flexibility for various promising applications.

## Abstract 2

A 3D-rGO wrinkled film with a graphene expansion structure and stretchable 3D-rGO bulge for application in multi-functional sensors is reported.

## Graphical abstract

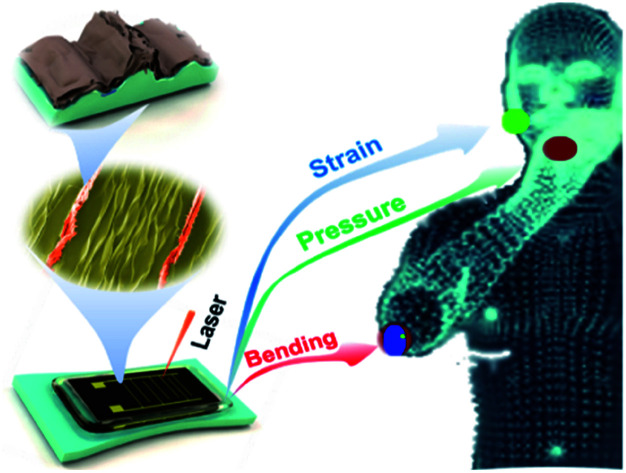

## Introduction

Wearable electronic devices used in the human body mainly focus on electro-mechanical conversion.^1^ In general, the main principle is to convert the changes caused by motion into the output of electrical signals.^2–5^ The significant functional advantages of smart wearable devices have become the focus of attention, among which multi-functional design has become an important strategy to ease the burden on participants in the medical service.^6–9^ Fundamentally, the structural design of the active sensing layer in electromechanical sensors is an effective and straightforward implementation method.^10–14^ Designing and constructing suitable structures are not only beneficial for offsetting the rigidity and brittleness of materials, but can also be used to trigger more accurate electrical responses and improve their sensitivity.

Recently, graphene has been widely used in electrochemical sensing, photoelectric conversion and electromechanical sensing^15–18^ owing to its superior electroconductivity, high thermal conductivity and great mechanical strength.^19–22^ Compared to graphene, graphene oxide (GO), which is the derivative of graphene, has fascinating properties for a functional design. Due to the hydrophilic nature of GO, it is facile to form a uniform film with ordered stacking sheets.^23–25^ As a result, the fabrication of GO films has become a straightforward approach, which can be achieved *via* various methods, such as evaporation and vacuum filtration.^26,27^ For example, Qu *et al.* developed a highly ordered GO nanolayer film for moist-electric conversion through asymmetric humidity, taking advantage of the solvent evaporation induced self-assembly of GO solution.^28^ However, because of the inherent insulation of GO, which limits certain applications, it is necessary to acquire great conductance by reducing GO sheets for further sensing applications.^29^ Among the various reduction technologies, *e.g.*, using reducing agents such as hydrazine hydrate, hydroiodic acid, and other chemical reagents, hydrothermal reduction, high temperature thermal reduction and laser reduction,^30–34^ laser reduction is the most friendly, convenient and efficient method for reducing GO.

Structural design provides greater added value to advanced functional materials. Naturally, a wrinkle structure is a type of macroscopic three-dimensional structure, which possesses the characteristics of a large specific surface area and variable range of aspect ratios,^35^ and can be obtained by shrinkage under heat condition, non-uniform solvent evaporation and releasing of the pre-stretching matrix.^36–38^ Moreover, the specific parameters of wrinkles, such as their size, direction, gradient change and ridge curvature, can be controlled by adjusting the thickness, the stretching ratio of the material and other aspects.^38,39^ Khine *et al.* devised a wrinkled CNT film as the active layer in sensors, endowing the CNT film with great flexibility, which could be applied on the human body buckling skin to detect physiological signals.^40^ In general, wrinkles can greatly improve the flexibility and stretchability of rigid active materials. Thus, many researchers have focused on active material-based wrinkle structures. Recently, our group reported a gradient GO wrinkle with hierarchical structures for stretchable pressure sensors, which achieved a high sensing factor of 178 kPa^−1^.^34^ However, the special design of ridges to introduce new functions or improve the original properties of materials has not been explored to date. The advantages of wrinkled structures in flexible sensing applications also need to be investigated.

Considering the structural design based on the fabrication technique, the effect of laser technology on carbon-based materials enables the preparation of surfaces with a 3D structure. Tour *et al.* developed laser-induced graphene (LIG) technology to prepare 3D graphene *in situ* on carbon-based materials such as commercial polyimide and food.^41,42^ This type of technology can achieve the simultaneous regulation of the chemical structure and physical structure of carbon-based materials, and can be used to obtain microstructure protrusions on the surface of materials by regulating the laser conditions.^43,44^ Recently, functionalization has been achieved using laser-induced techniques on various carbon-based materials for broad applications, such as in wearable human body detection and electrocatalysis.^45–48^ However, for rigid active materials, the single laser-induced technique still has limitations in the preparation of stretchable 3D structural films. Hence, high-sensitivity sensors with a stretchable 3D architecture still need to be further explored.

Therefore, in this work, we proposed a new method named pre-straining & laser reduction (PS&LR), in which a pre-straining force induces the formation of a 3D architecture followed by modification of the physicochemical properties of stacked GO sheets with a laser. This technology combines the advantages of bionic wrinkles on flexibility and the advantages of laser treatment on the efficient reduction of GO. PS&LR was successfully used for the preparation of 3D-rGO bulge-based wrinkled GO films with multilevel micropores. The prepared films possessed a special 3D microstructure, which enables different types of sensing devices to be fabricated, namely, sensors responsive to bending, pressure, and tensile responses. The resulting sensors maintained high response and sensitivity. The synergistic principle of the wrinkle force and laser scanning was explored in detail.

## Results and discussion

**Fig. 1:**
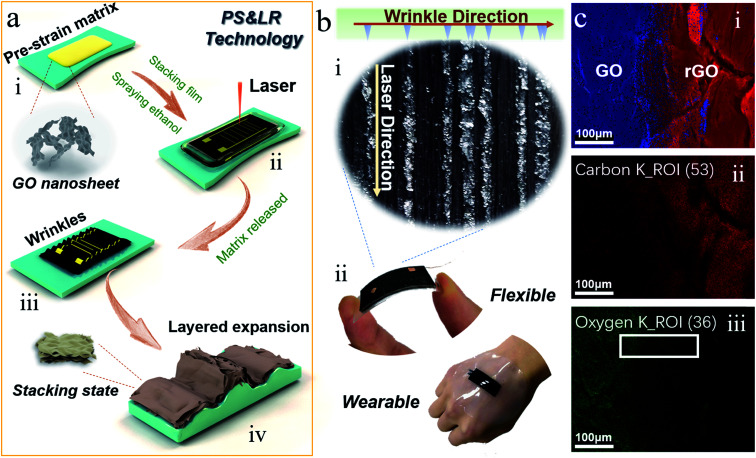
Fabrication process and elemental analysis characterization of the wrinkled GO film with 3D-rGO bulge. (a) Fabrication process. (b) Photograph of 3D-rGO film and flexible sensor. (c) EDX mapping images of the GO and rGO area, (i) total spectrum of elements O and C, (ii) element C, (iii) element O.

The fabrication process of the wrinkled GO film with high 3D-rGO bulges is shown in Fig. 1a. First, GO was coated on a pre-strained substrate (VHB, 3M Company, 3M5604) with series of pre-strain rates (Fig. 1a(i)), which were denoted ad GO-VHB. The GO-VHB film was scanned under the laser device with a designated program (Fig. 1a(ii)). Subsequently, the pre-strain was released, and the film was put into an oven for the relaxation step (Fig. 1a(iii)). The final structure with a high 3D-rGO bulge is shown in Fig. 1a(iv). There are a large number of lower regular wrinkles on both sides of the 3D-rGO bulge. The circuit connection was realized using silver paste and copper wire, in which ITO film was used as the counter electrode for the pressure device. The device for body application was encapsulated by liquid silicone. The photograph of the prepared 3D-rGO film and the physical display of the sensing film are shown in Fig. 1b. As shown in Fig. 1b(i), apparent differences can be found under the PS&LR method. Moreover, the sensing film has superior flexibility derived from its wrinkled structure (Fig. 1b(ii)).

Aiming to assess the reduction effect and element change resulting from the laser process, EDX mapping of the area containing GO and rGO was performed. The results of the element analysis on both sides of GO–rGO are presented in Fig. 1c. From the total spectrum in Fig. 1c(i), it is apparent that the relative content ratio of O on the GO side is much larger than that on the rGO side (blue part, referring to O). Comparing the C and O parts, the relative content ratios of O and C on both sides can be observed more clearly. Furthermore, the XPS spectra of GO and rGO are shown in the ESI (Fig. S1†), which demonstrated the differences in the C and O content. In particular, on both sides of the boundary between GO and rGO in Fig. 1c(ii), the fluorescence density of C on the rGO side is significantly larger than that on the GO side. In Fig. 1c(iii), the fluorescence intensity of the O on the rGO side is significantly lower than that on the GO side. This also clearly shows the reduction effect of the laser on the rGO side. Additionally, what is in particular interesting is that there is a clear trend of gradient fluorescence intensity from rGO to GO (as shown in the boundary of both sides, white rectangular area), which reflects a microscopic feature of laser reduction. We also characterized the functional groups of the rGO and GO, as shown in the ESI (Fig. S2,† FTIR spectra). The representative and strong absorption peaks in GO include the absorption peak generated by the stretching-vibration of –OH (near 3233 cm^−1^), the GO edge peak (near 2929 cm^−1^) and the stretching-vibration absorption peak of –C=O and C–OH (near 1052 cm^−1^). Compared to the absorption peaks determined for GO, there is a significant decrease in the intensity of these peaks in the spectrum of rGO. The reduction effect of the oxygen-containing groups inside and outside the GO layer by the laser is also illustrated.

**Fig. 2:**
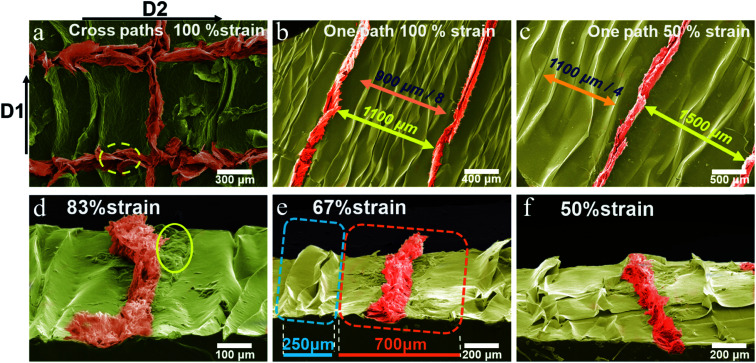
Pseudo-color SEM images of the wrinkled GO films with 3D-rGO bulges. (a) Top view of the 3D-rGO film in the condition of the laser scanning path in two different directions. Top view of 3D-rGO films with different stain ratios in the condition of the laser scanning path in one direction of (b) 100% and (c) 50%. Side inclination view of the 3D-rGO films with different strain ratios of (d) 83%, (e) 67%, (f) 50%.

Scanning electron microscopy (SEM) was used to observe the 3D-rGO wrinkled film. As a means to facilitate the observation of the structure, pseudo-color processing was performed on the SEM images to analyze the features of the structure more clearly. The results are shown in Fig. 2. Firstly, the results of the correlational analysis between the laser path and the wrinkle direction are displayed in Fig. 2a. The laser scanned along the direction of stretching (*D*2) and the direction of the wrinkle ridge (*D*1). It can be seen that the 3D architecture formed by PS&LR in the direction of the wrinkle is readily distinguishable. A significant expansive 3D architecture appears along *D*1. Relatively, the 3D-rGO is larger than the GO wrinkle ridge in the dimension perpendicular to the surface of the film. Apparently, it can be found that the 3D architecture is rougher, which is considered to have a positive effect of improving the sensitivity in various stress sensing studies. However, the structure along *D*2 is wider than that along *D*1, and a petal-like structure appeared in the former (the yellow dotted line in Fig. 2a). This is due to the effect of the rGO layer being compressed by the pre-straining force. Interestingly, the above findings provide a greater possibility for the application of laser technology in the preparation of stretchable wrinkled films with special 3D-rGO structures. The innovative method of using PS&LR to fabricate a stretchable 3D-rGO architecture provides rGO as an active sensing layer with greater advantages, and the 3D bulge film has greater flexibility and stretchability than the laser-reduced GO film, as demonstrated below.

Next, the structure in the *D*1 direction was explored further. As shown in Fig. 2b and c, two types of 3D bulge films were prepared under the conditions of two strain ratios (100% and 50%), direction *D*1 and uniform laser parameters, respectively. It can be clearly found that the density of the GO wrinkles and 3D-rGO bulge under 100% strain ratio was greater than that for the case of 50% strain ratio. Observing the 3D-rGO in each laser path alone, it can be seen that the size is significantly different from the nearby GO wrinkles, and a relatively flat GO region with a certain width appears on both sides of the 3D-rGO. Owing to the significant difference in mechanical strength and microstructure between rGO and GO, the shrinkage force is dispersed during the excessive bulging of 3D-rGO. As a result, the flexible matrix and GO film exhibited no wrinkles in a certain range.

The morphological structure under the angle of inclination is shown in Fig. 2d–f. Comparing the 3D-rGO bulges of the three samples, it is visible that as the strain ratio decreased, the height of the expansive structure gradually decreased. Additionally, as can be seen from Fig. 2d, some GO regions with a non-uniform morphology (yellow circle region in Fig. 2d) can be found on the sides of 3D-rGO, which is mainly due to the change in the thermal environment, in which the laser focus spot extends outward. Furthermore, from the two dotted areas in Fig. 2e, it can be quantitatively analyzed that the width of the GO wrinkle is narrow (about 83 μm per ridge), depending on the matrix shrinkage force. The area formed by a single 3D-rGO bulge is wider (about 700 μm). Differently, the width is determined by the effect of laser reduction (*i.e.*, the change in rGO mechanical properties brought by laser) and the total matrix shrinkage force in the region. In terms of shrinkage force alone, the bulging process of a single 3D-rGO bulge is subject to the total force in the wide region, which also results in a larger size than the nearby GO wrinkles. As the strain ratio reduces, the shrinkage force declines so that the 3D-rGO bulge height also reduces.

**Fig. 3:**
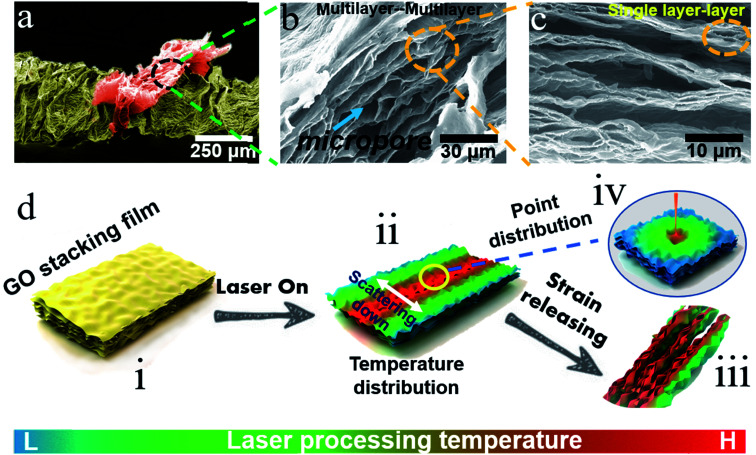
Mechanism model for illustrating the principle of structure formation under the new method and the SEM topographical images of the 3D-rGO bulge structure at different magnifications. (a) Pseudo-color SEM image of 3D-rGO bulge. (b) SEM image of multilayer–multilayer micropores. (c) SEM image of single-interlayer micropores. (d) PS&LR principle, (i) GO film, (ii) temperature distribution under the laser process, (iii) multi-level pore structure in three dimensions, (iv) degree distribution of reduction in the focused point of the laser.

The microstructure of 3D-rGO as the active sensing layer is a major factor governing the performance of the sensor. Thus, to further explore the principle of structure formation under this innovative method (*i.e.* PS&LR) and explore the sensor applications, the microstructure of 3D-rGO was characterized. Combining the above findings a mechanism model was established, as shown in Fig. 3.

Firstly, Fig. 3a–c present the SEM topographical images at different magnifications. In addition to the greatly expansive 3D-rGO bulge structure shown in Fig. 3a, there are a large number of micropores inside the 3D-rGO bulge, as shown in Fig. 3b. These large micropores exist between the laminated rGO (multilayer) layers. It is believed that the micropore structure between the multilayers provides greater variability in the conductance of the active layer. Interestingly, as shown in Fig. 3c, the most striking observation was that many subtle oval micropores appeared between the single-layer rGO in many rGO multilayers. These results provide important insights that the 3D-rGO expandable bulge with multi-level pore structure can bring variability to the multifunctional design of sensors. Correspondingly, the flexible strain sensor can be considered to utilize the pore expansion–compression between the interlayers for sensing design, and flexible pressure sensors can be designed to work with the reversible deformation of the 3D expansion bulge and the interlayer pores.

As mentioned above, the fabrication method based on PS&LR is relatively simple and easy to control and implement. To illustrate the principle of structure formation, the GO layer film was further investigated. In general, a tightly packed GO film produces a certain range of rGO in the laser path of the wrinkle formation direction (the red area of the temperature distribution is the main reduction area, see Fig. 3d(i) and (ii)). As shown in Fig. 3d(iv), the focused point of the laser is regarded as a gradual distribution circle whose heat gradually decreases from the inside to the outside. Therefore, the heat distribution formed on the laser path is as shown in Fig. 3d(ii), declining on both sides. The laser reduction of the GO layer can be deemed to be a heat-assisted reduction, and thus the degree of reduction of GO from the inside to the outside is also a gradient distribution. The multilayer pore structure shown in Fig. 3a–c obtained in this process also exhibited a gradient distribution, decreasing until the rGO region disappeared. This gradient effect led to the formation of interlayer pores and mechanical strength in the reduction region. Based on the above effects of the laser, the strain release of the flexible substrate generated the heavier stress compression in the reduction region compared to other regions not affected by the laser (*i.e.*, forming a conventional GO wrinkle region). In summary, due to the expansion of the structure and more pores appearing in the rGO interlayer, the wrinkled force can be more dispersed toward the rGO region. As a result, the force effect is more significant contributing to shape the high 3D-rGO bulge with multi-level micropores (see Fig. 3d(iii)).

**Fig. 4:**
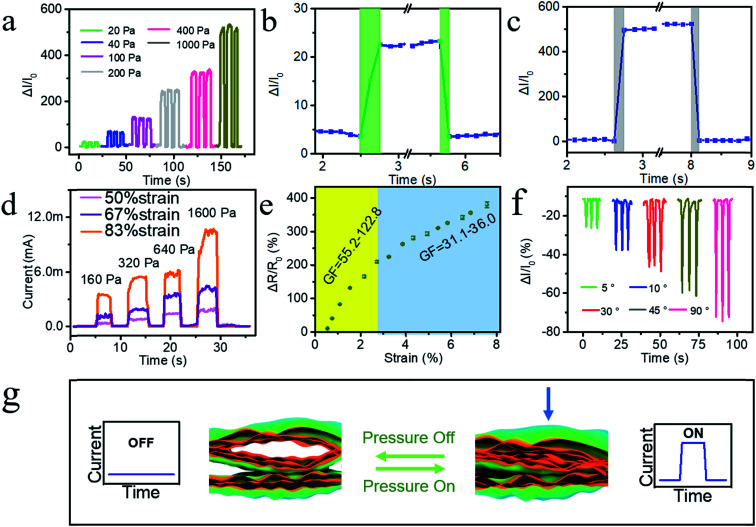
Electrical performance characterization of the three types sensing films and the response mechanism of the tensile sensor and the bending stress sensor. Electrical performance curves of the pressure sensor: (a) response to different pressure, (b) response and recovery time under 20 Pa, (c) response and recovery time under 1000 Pa, and (d) response of the three pressure sensors with different pre-strain ratios to different pressures. (e) Response of the stress sensor under different strain ratios ranging from 0% to 7.8%. (f) Response of the bending sensor under different bending angles ranging from 5° to 90°. (g) Schematic diagram of the response mechanism of the tensile sensor and the bending stress sensor.

The above analysis of the 3D-rGO bulge structure reveals the 3D-rGO wrinkle GO film possesses the characteristics of versatility in comparison to the single functional design of conventional active layer films. For example, due to the low stretchability of laser-reduced GO films, they can only be used as a supercapacitor or a stress sensor with a low strain ratio. Therefore, to demonstrate the uniqueness of this approach and its suitability for applications, the electrical performance of three types of sensors was investigated, including pressure sensors, tensile sensors and bending sensors. Generally, the 3D-rGO conductive path of different forms is designed on the surface of the GO film by using the design principle of the optical path program. The test results are shown in Fig. 4.

Fig. 4a shows the pressure sensing result, presenting the relative current response curve of the sensor under different pressures and reflecting its responsiveness to pressure. It can be found that the relative current signal exhibited an increasing trend with irregular steps as the stress increased, and had a large difference in the relative current signal. The relative current signal changed greatly under high pressure (1000 Pa), but the signal also began to appear disordered. The results may be explained by the fact that the 3D-rGO expansion bulge becomes deformed when subjected to pressure. Especially, when the pressure is small, the current change mainly originates from the contact area between the irregular section of the expansion bulge and the counter electrode. As the pressure increases continually, it is mainly the compression between the multi-level pore contributing to the current change. Until the pressure is too high, the 3D-rGO shows a large macroscopic deformation, and thus the counter electrode and the 3D-rGO bulge surface begin to come into contact.

In addition, the response–recovery time of the pressure sensor under low stress and high stress was determined. As shown in Fig. 4b and c, under low pressure (20 Pa), the response time of the sensor was about 60 ms and the recovery time was about 30 ms. In contrast, under high stress (1000 Pa), the time was about 30 ms and 25 ms, respectively. Generally, the response–response was short under high pressure, which is advantageous in sensor applications and can bring a timely signal feedback. The relative hysteresis of response time under low pressure is mainly due to the large contactless space caused by the irregularity of the top. To clearly show the performance, the corresponding time history of the input load in Fig. 4a–c is shown in Fig. S3.†

Aiming to investigate the influence of strain ratio, the electrical performance of three prepared films with different strain ratios was characterized, as shown in Fig. 4d. As the size of the 3D-rGO bulge structure increased (the stretching ratio gradually increases), the signal intensity increased significantly mainly due to the fact that the increase in height resulted in more accessible deformation ability and variable space for the 3D-rGO bulge.

Then, the electrical performance associated with tensile is shown in Fig. 4e. As the strain ratio increased, the relative resistance change was gradually reduced. Before the ratio of 2.7%, the sensitivity factor was 55.2–122.8, and when the ratio was greater than 2.7%, the sensitivity factor was 31.1–36.0. Taken together, the overall performance became poor gradually. It is worth noting that this variation is different from the variation of the curve exhibited by the tensile sensor with the traditional microcrack design, in which the curve shows a positive slope. The negative slope exhibited by the 3D-rGO bulge-based wrinkled GO film is quite unique. According to our analysis, the main reason for this is that the 3D-rGO fabricated by PS&LR possesses macroscopic large-sized bulges perpendicular to the film plane, in which there exists an expansion effect parallel to the film plane. As a result, when the film is under stress, these factors contribute stress dispersion to the film without influencing the conductance path in the 3D-rGO. Considerably, the multilevel pore structure provides advantages for high sensitivity factor and the 3D-rGO bulge provides more space for stretching to disperse the stress.

Fig. 4f shows the electrical performance curve of the bending sensor. The positive bending strategy (indicating positive bending, as presented in 3D-rGO on the outside of the curved edge) was adopted with four different angles of 5°, 10°, 30°, 45°, and 60°. As the bending angle increased, the relative current value (negative) gradually decreased. As observed from the characteristics of the curve, the sensor maintained excellent ability for the bending-recovery response and great repeating performance. It is worth noting that due to the small stretching ratio parallel to the film, the response mechanism of the bending response relative to the tensile response mainly relies on the deformation of the multilevel pores.

Fig. 4g illustrates the consistent response mechanism of the tensile sensor and the bending stress sensor, which is different from the pressure sensor, as reported above. The key point of the mechanism mainly focuses on the change in the interlayer contact caused by the change in the 3D-rGO multi-level pore structure under external pressure. However, the response mechanism of the pressure sensor is based on the size change of both the bulge and micropores.

**Fig. 5:**
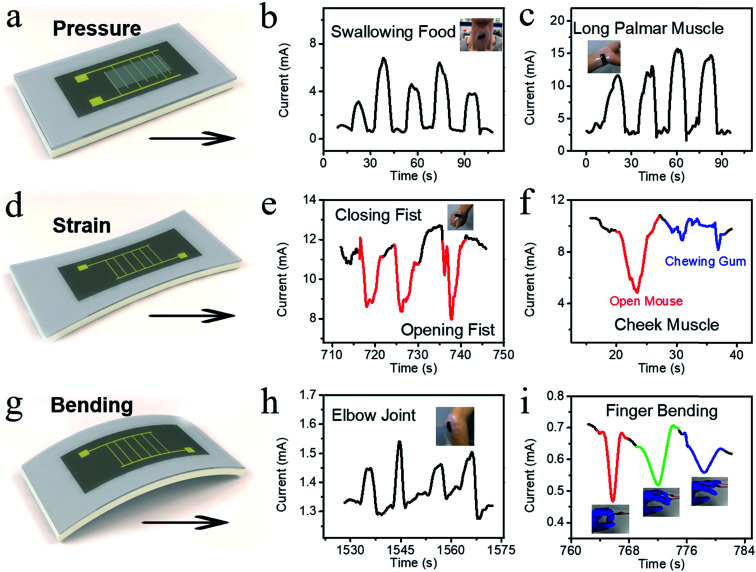
Multi-functional application exploration on the human body based on the unique structures. Pressure-sensing film for human body application: (a) laser design and construction of the pressure sensor, (b) recoding of the throat movement for swallowing food, and (c) movement of the long palmar muscle. Tensile-sensing film for strain application: (d) laser design and construction for the strain application, (e) negative current change for the closing movement of the palm, and (f) current change for opening the mouth and chewing gum. Tensile-sensing film for bending application: (g) laser design and construction for the bending application, (h) movement of the elbow joint, where the sensor was attached for the inner bending, and (i) movement of the finger bending, where the sensor was attached for the outer bending.

Based on the above-mentioned electrical performance characterization results and sensing mechanism analysis as well as the scope of application of different sensors, three strategies were applied in the fabrication of sensors for distinct parts of the human body. The first one is the pressure sensor shown in Fig. 5a. Fig. 5b and c present the applications on the human body. We directly applied the pressure sensor to the throat for detecting the physical swallowing motion (Fig. 5b). It can be seen that the different intensities of stress compression caused by swallowing on the sensor could be clearly recorded according to the distinguished current–pressure curve. In addition, Fig. 5c shows the application on the tendon movement of the wrist, which could detect the up and down movements of the tendon.

The tensile sensor applications are shown in Fig. 5d–f, among which Fig. 5d is the laser design and construction of the tensile sensor. Based on the multilevel pore structure and 3D-rGO bugle-based wrinkled GO, we applied the tensile sensor to the expansion and contraction of the epidermis. As shown in Fig. 5e, the sensor film was attached to the epidermis of the palm. When the palm opened and closed, the sensor could recognize the movement of the epidermis. According to the current signal, the sensor exhibited a signal showing the current value increased negatively as the palm closed. This is due to the fact that the multilevel pore structure inside the 3D-rGO and the bulge attached to the substrate appeared to relax. In addition, the sensor was implemented on the muscles of the cheeks to detect the stretching and contraction of the facial muscles during chewing (Fig. 5f). As a result, the muscle movements of opening the mouth (red) and chewing (blue) could be obviously distinguished.

Finally, we applied the sensor for bending tests. Fig. 5g shows the laser design and construction of the tensile sensor. Based on the sensing characteristics, two strategies were implemented to display the advantage extended by PS&LR. One is the inner bending mode (the 3D-rGO layer is on the inside of the curve), as shown in Fig. 5h. The sensor was attached to the elbow joint (initially bending) to detect the rapid-slow bending of the elbow, which utilizes the compression of the micropore. The other is the outer bending mode (the 3D-rGO layer is on the outside of the curve), as shown in Fig. 5i. The sensor was applied to the finger joints to recognize different degrees of finger bending. Inevitably, due to the difference in sensor types prepared in separate batches, the signal intensity displayed some subtle differences, which have little influence on the application of the detection function. Through the above application and exploration, it is proven that this type of flexible and stretchable sensing device with the 3D-rGO structure can realize multi-functional applications without excessively complicated circuit design and device assembly.

## Conclusions

In summary, the PS&LR method was established in this work for the first time. This concept combines and highlights the advantages of both wrinkled structures on flexibility and laser on the efficient reduction of GO. Especially, the stretchable sensing wrinkled GO film with a 3D-rGO expansion bulge was prepared efficiently, in which internal multilevel micropore structures were distributed and native stretchable ridges existed. Based on this unique structure, some types of sensing films for different detection modes were fabricated, showing great distinguishing abilities. The unique structures endow the sensing film a large design space for application in fields involving various sensing mechanisms. In particular, the mechanisms of pressure and tensile sensing applications are different, but the films were capable of balancing both types of mechanisms. One is that the pressure sensing device can be designed based on the compressibility of the 3D-rGO bulge in the direction perpendicular to the film, and in the other, the tensile sensing film can be designed by using the programmatic laser path based on the scalability of the 3D-rGO internal micropores and the wrinkles in the direction parallel to the film. Briefly, we developed a methodology that provides a platform for the preparation of 3D-rGO films with potential multi-functional applications.

## Experimental

### Preparation of GO dispersion

The GO dispersion was prepared using the modified Hummers' method reported in our previous work.^49^

### Preparation of uniaxially stretched GO film

VHB tape (3M Company, 3M5604) was cut into a rectangle of 5 cm × 3 cm, and stretched using a QGG manual stretching platform. The pre-stretching ratios ranged from 50% to 100%. The draw ratio was maintained for 5 min to ensure the molecular chain creep to the end. Then a transparent tape was used to limit a 3 cm × 5 cm rectangular area at the edge of the pre-stretched VHB tape. 3 mg mL^−1^ GO dispersion (2 mL) was dropped on the stretched VHB tape with a region (3 cm × 3 cm) limited by commercial transparent tape. Owning to the large surface tension between the dilute solution and the transparent tape, a uniform horizontal level was present inside the solution. Once the dispersion level was stable, the entire assembly was placed in a constant temperature oven of 50 °C until the GO stacked film was obtained.

### Preparation of the 3D-rGO-based wrinkled GO film

The surface of the GO film prepared above was suitably wetted with 1 mL ethanol and covered with a transparent glass slide. The GO film was reduced by a custom laser device (semiconductor laser) with a focal length of 100 cm, power of 4.5 W and moving rate of 200% (2 cm s^−1^). The laser reduction path was determined by a program programmed with custom software. Thereafter, the pre-stretching ratio was released immediately. Then, the manual stretching platform was placed in a constant temperature oven of 50 °C for 30 min to ensure that the flexible substrate recovered completely. Finally, the desired 3D-rGO bulge-based wrinkled GO film was obtained. The prepared film was cut into pieces for subsequent characterization and other tests. Here, the sensor for human body application was encapsulated by liquid silicone for room-temperature curing (commercial HY-9060).

### Material characterization

The surface structure was observed by scanning electron microscopy (Hitachi S-4700, acceleration voltage of 20 kV). Energy dispersive spectroscopy of the SEM images was obtained using a Hitachi S-4800 at an acceleration voltage of 15 kV, equipped with an energy dispersive X-ray spectrometer. Chemical characterization was performed *via* Fourier transform infrared spectroscopy (Bruker Vertex 70v, wavenumber range of 600 to 4000 cm^−1^, 2 cm^−1^ resolution, averaging 32 scans) and XPS spectra were collected using an ESCALAB 250 equipped with monochromatic Al KR 150 W (200 eV for survey, 30 eV for high-resolution scans).

### Device characterization

Pressure sensor measurements were performed using a precision DC power supply (Keithley 2280S-60-3) with relative software to record the current data. The pressure calculation was based on the weight and area. The strain-response performance was monitored by the precision DC power supply and QGG stretching platform with manual stretching referring to the distance recording tool. The bending response performance was assessed by the precision DC power supply to record the current data while the sensor was attached on human skin or thin PET film was bent at a series of angles by writing on paper.

## Conflicts of interest

There are no conflicts of interest to declare.

## Supplementary Material

NA-001-C9NA00429G-s001

## References

[cit1] Zang Y., Zhang F., Di C.-A., Zhu D. (2015). Mater. Horiz..

[cit2] Lv L., Zhang P., Xu T., Qu L. (2017). ACS Appl. Mater. Interfaces.

[cit3] Pang Y., Tian H., Tao L., Li Y., Wang X., Deng N., Yang Y., Ren T. L. (2016). ACS Appl. Mater. Interfaces.

[cit4] An R., Zhang B., Han L., Wang X., Zhang Y., Shi L., Ran R. (2019). J. Mater. Sci..

[cit5] Luo N., Zhang J., Ding X., Zhou Z., Zhang Q., Zhang Y.-T., Chen S.-C., Hu J.-L., Zhao N. (2018). Adv. Mater. Technol..

[cit6] Hong S. Y., Oh J. H., Park H., Yun J. Y., Jin S. W., Sun L., Zi G., Ha J. S. (2017). NPG Asia Mater..

[cit7] Yamamoto Y., Harada S., Yamamoto D., Honda W., Arie T., Akita S., Takei K. (2016). Sci. Adv..

[cit8] Kanao K., Nakata S., Arie T., Akita S., Takei K. (2017). Mater. Horiz..

[cit9] Parida K., Kumar V., Wang J., Bhavanasi V., Bendi R., Lee P. S. (2017). Adv. Mater..

[cit10] You I., Choi S.-E., Hwang H., Han S. W., Kim J. W., Jeong U. (2018). Adv. Funct. Mater..

[cit11] Wang C., Wang C., Huang Z., Xu S. (2018). Adv. Mater..

[cit12] Shuai W., Peng X., Yun L., Zhang J., Tao C. (2018). J. Mater. Chem. C.

[cit13] Wan Y., Qiu Z., Ying H., Yan W., Zhang J., Liu Q., Wu Z., Guo C. F. (2018). Adv. Electron. Mater..

[cit14] Duan G., Hong F., Huang C., Jiang S., Hou H. (2018). J. Mater. Sci..

[cit15] Ren H., Tang M., Guan B., Wang K., Yang J., Wang F., Wang M., Shan J., Chen Z., Wei D., Peng H., Liu Z. (2017). Adv. Mater..

[cit16] Lin H., Sturmberg B. C. P., Lin K.-T., Yang Y., Zheng X., Chong T. K., de Sterke C. M., Jia B. (2019). Nat. Photonics.

[cit17] Mo R., Li F., Tan X., Xu P., Tao R., Shen G., Lu X., Liu F., Shen L., Xu B., Xiao Q., Wang X., Wang C., Li J., Wang G., Lu Y. (2019). Nat. Commun..

[cit18] Tao L. Q., Zhang K. N., Tian H., Liu Y., Wang D. Y., Chen Y. Q., Yang Y., Ren T. L. (2017). ACS Nano.

[cit19] Yu X., Cheng H., Miao Z., Yang Z., Qu L., Shi G. (2017). Nat. Rev. Mater..

[cit20] Rezapour M. R., Myung C. W., Yun J., Ghassami A., Li N., Yu S. U., Hajibabaei A., Park Y., Kim K. S. (2017). ACS Appl. Mater. Interfaces.

[cit21] Pan K., Fan Y., Leng T., Li J., Xin Z., Zhang J., Hao L., Gallop J., Novoselov K. S., Hu Z. (2018). Nat. Commun..

[cit22] Khrapach I., Withers F., Bointon T. H., Polyushkin D. K., Barnes W. L., Russo S., Craciun M. F. (2012). Adv. Mater..

[cit23] Wu J., Li H., Qi X., He Q., Xu B., Zhang H. (2014). Small.

[cit24] Shao Y., Wang H., Zhang Q., Li Y. (2014). NPG Asia Mater..

[cit25] Zhou K. G., Vasu K. S., Cherian C. T., Neek-Amal M., Zhang J. C., Ghorbanfekr-Kalashami H., Huang K., Marshall O. P., Kravets V. G., Abraham J., Su Y., Grigorenko A. N., Pratt A., Geim A. K., Peeters F. M., Novoselov K. S., Nair R. R. (2018). Nature.

[cit26] Zhang W., Wang L., Sun K., Luo T., Yu Z., Pan K. (2018). Sens. Actuators, B.

[cit27] Wang M., Qiu Y., Jia J., Wang C., Deng J., Pan K. (2019). Adv. Mater. Technol..

[cit28] Qu L., Yuan L., Fei Z., Cheng Z., Deng Y., Xiao Y., Cheng H., Zhang P., Huang Y., Hui B. S. (2018). Energy Environ. Sci..

[cit29] Compton O. C., Nguyen S. T. (2010). Small.

[cit30] Pei S., Cheng H.-M. (2012). Carbon.

[cit31] Zhuo Q., Jing G., Peng M., Bai L., Deng J., Xia Y., Ma Y., Zhong J., Sun X. (2013). Carbon.

[cit32] Dreyer D. R., Todd A. D., Bielawski C. W. (2009). Chem. Soc. Rev..

[cit33] Zhou Y., Bao Q., Varghese B., Tang L. A., Tan C. K., Sow C. H., Loh K. P. (2010). Adv. Mater..

[cit34] Jia J., Huang G., Deng J., Pan K. (2019). Nanoscale.

[cit35] Hu H. W., Haider G., Liao Y. M., Roy P. K., Ravindranath R., Chang H. T., Lu C. H., Tseng C. Y., Lin T. Y., Shih W. H., Chen Y. F. (2017). Adv. Mater..

[cit36] Deng S., Berry V. (2016). Mater. Today.

[cit37] Liu W., Liu N., Yue Y., Rao J., Cheng F., Su J., Liu Z., Gao Y. (2018). Small.

[cit38] Jianfeng Z., Seunghwa R., Nicola P., Qiming W., Qing T., Buehler M. J., Xuanhe Z. (2013). Nat. Mater..

[cit39] Wenzhong B., Feng M., Zhen C., Hang Z., Wanyoung J., Chris D., Chun Ning L. (2009). Nat. Nanotechnol..

[cit40] Park S. J., Kim J., Chu M., Khine M. (2016). Adv. Mater. Technol..

[cit41] Chyan Y., Ye R., Li Y., Singh S. P., Arnusch C. J., Tour J. M. (2018). ACS Nano.

[cit42] Li L., Zhang J., Peng Z., Li Y., Gao C., Ji Y., Ye R., Kim N. D., Zhong Q., Yang Y. (2016). Adv. Mater..

[cit43] Stanford M. G., Yang K., Chyan Y., Kittrell C., Tour J. M. (2019). ACS Nano.

[cit44] Luong D. X., Yang K., Yoon J., Singh S. P., Wang T., Arnusch C. J., Tour J. M. (2019). ACS Nano.

[cit45] Xie Y., Zhang C., Su J.-W., Deng H., Zhang C., Lin J. (2019). ChemSusChem.

[cit46] Dosi M., Lau I., Zhuang Y., Simakov D. S. A., Fowler M. W., Pope M. A. (2019). ACS Appl. Mater. Interfaces.

[cit47] Tao L.-Q., Tian H., Liu Y., Ju Z.-Y., Pang Y., Chen Y.-Q., Wang D.-Y., Tian X.-G., Yan J.-C., Deng N.-Q., Yang Y., Ren T.-L. (2017). Nat. Commun..

[cit48] Nayak P., Kurra N., Xia C., Alshareef H. N. (2016). Adv. Electron. Mater..

[cit49] Wang J., Zhang P., Liang B., Liu Y., Xu T., Wang L., Cao B., Pan K. (2016). ACS Appl. Mater. Interfaces.

