# Supplementary material for: Multi-functional stretchable sensors based on a 3D-rGO wrinkled microarchitecture
Source: Nanoscale Adv. 2019 Sep 27;1(11):4406–14. doi: 10.1039/c9na00429g (PMC9419508; doi:10.1039/c9na00429g)
Supplement: NA-001-C9NA00429G-s001 [file NA-001-C9NA00429G-s001.pdf]

Electronic Supplementary Material (ESI) for Nanoscale Advances.
This journal is © The Royal Society of Chemistry 2019

Supporting Information

Multi-functional Stretchable Sensors Based on 3D-rGO Wrinkled Microarchitecture

*Jin Jia, † Jianping Deng, *† and Kai Pan*†*

†College of Materials Science and Engineering (CMSE), Beijing University of Chemical Technology,

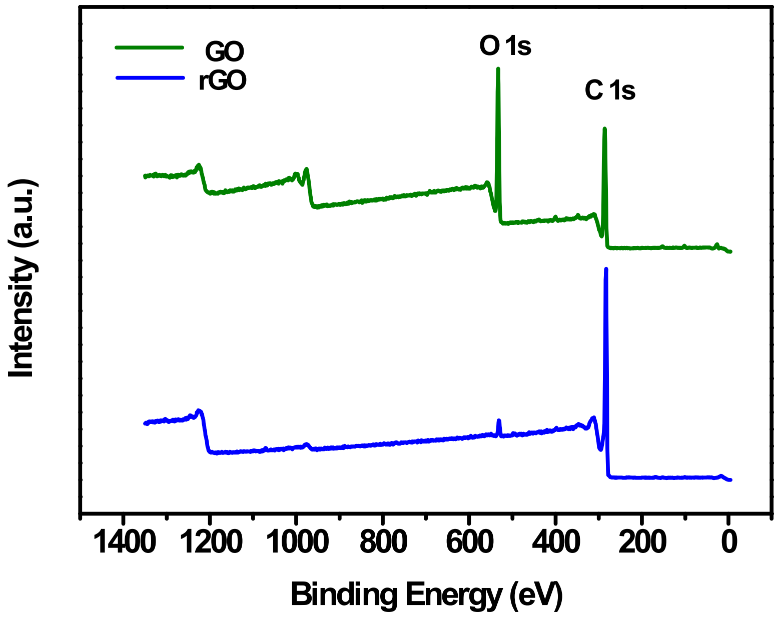

Chaoyang District North Third Ring Road 15, Beijing 100029, China

Figure S1. Survey XPS spectra of GO (before reduction) and rGO (after reduction).

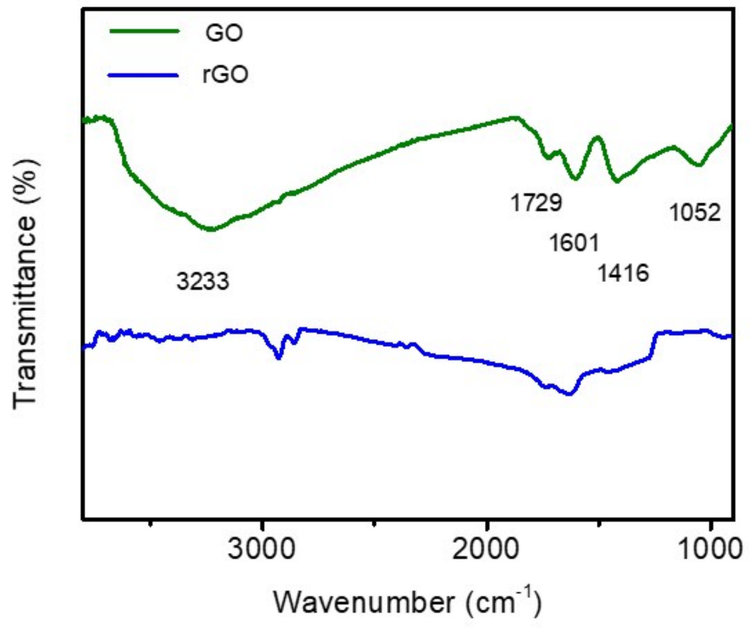

Figure S2. FTIR spectra of GO (before reduction) and rGO (after reduction).

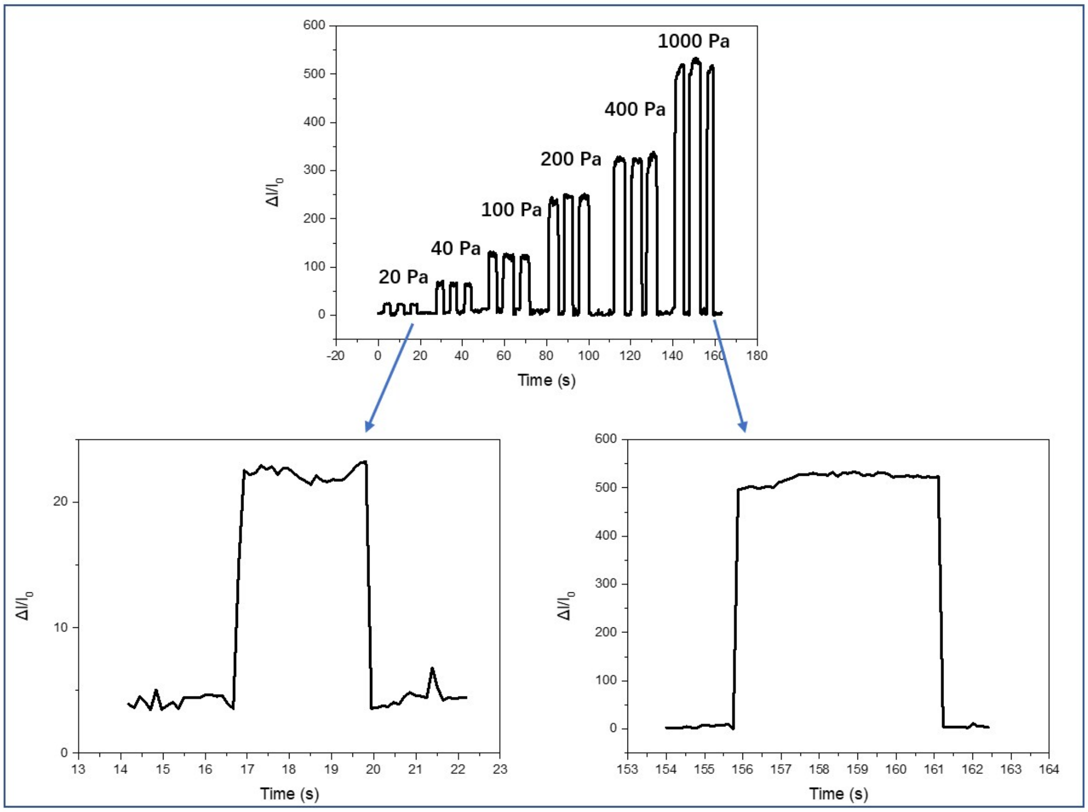

Figure S3. The time history of the input load in Fig. 4 a, b, c.
